# The Scientific American

**Published:** 1895-12

**Authors:** 


### The Scientific American.

This unrivalled periodical, now in its fiftieth year, continues to maintain its high reputation for excellence, and enjoys the largest circulation ever attained by any scientific publication. Every number contains sixteen large pages, beautifully printed, elegantly illustrated; it presents in popular style a descriptive record of the most novel, interesting, and important advances in all the principal departments of science and the useful arts, embracing biology, geology, mineralogy, natural history, geography, archæology, astronomy, chemistry, electricity, light, heat, mechanical engineering, steam and railway engineering, mining, ship-building, marine engineering, photography, technology, manufacturing industries, sanitary engineering, agriculture, horticulture, domestic economy, biography, medicine, etc. A vast amount of fresh and valuable information pertaining to these and allied subjects is given, the whole profusely illustrated with engravings.

